# Navigating new spaces: the role of sports and leisure in the lives of Montréal’s immigrant and refugee communities

**DOI:** 10.3389/fpsyg.2026.1732261

**Published:** 2026-02-12

**Authors:** Can Özgider, Jordan Koch

**Affiliations:** 1 Department of Sports Management, Faculty of Sports Sciences, Canakkale Onsekiz Mart University, Canakkale, Türkiye; 2 Department of Kinesiology and Physical Education, Faculty of Education, McGill University, Montréal, QC, Canada

**Keywords:** language learning through recreation, refugee and immigrant integration, refugee wellbeing, social inclusion, sports and leisure participation

## Abstract

**Background:**

Migration poses significant challenges to individuals’ psychological wellbeing, identity, and social integration. Sport and leisure activities have been recognized as meaningful avenues for fostering coping, resilience, and a sense of belonging. Nevertheless, there is limited evidence on how newcomer adults—particularly refugees—experience and engage in these activities throughout their settlement process in Canada, and especially within the context of Montréal.

**Methods:**

This qualitative study examined how sport and leisure influenced the lives of 15 recent immigrants and refugees in Montréal, 12 of whom were refugees. Semi-structured interviews were conducted and thematically analyzed to identify barriers, experiences of exclusion, and strategies to promote integration.

**Results:**

Three key themes emerged: (1) socio-economic and structural barriers to leisure and sport participation, (2) experiences of social exclusion and marginalization in leisure spaces, and (3) combining sports and language learning to support inclusion and wellbeing. While socio-economic and linguistic barriers limited participation, some participants described sport and leisure as helpful for staying active, practicing French, and connecting with others during their early settlement.

**Conclusion:**

Findings suggest that sport and leisure function as ambivalent spaces for newcomers in Montréal—sites of both exclusion and potential empowerment. Integrating language learning into sport and leisure activities and reducing structural barriers may enhance social inclusion and support mental wellbeing. These insights highlight the importance of recognizing sport and leisure not merely as forms of physical activity or entertainment, but as vital psychosocial resources that contribute to settlement, resilience, and belonging in multicultural societies.

## 1 Introduction

International migration—the movement of individuals across national borders—has become a defining feature of the 21st century, driven by economic globalization, conflict, political instability, and security concerns (Potter et al., 2024; Bartram et al., 2014). Globally, there are 304 million international migrants, representing 3.7% of the world’s population, of whom approximately 42.7 million are refugees—people compelled to flee their home countries due to persecution, conflict, or violence (United Nations, 2025; UNHCR, 2025). Despite the scale of global displacement, research on refugee experiences, particularly their psychological and social wellbeing, remains limited.

Migration carries profound socio-economic and demographic consequences, but it also entails significant psychological impacts, including stress, trauma, identity reconstruction, and resilience challenges (Brance et al., 2024; Mwanri et al., 2022). Many migrants experience traumatic events—such as exposure to violence, forced displacement, or family separation—that shape mental health outcomes (Barua and Maheshwari, 2025). Upon arrival in host countries, newcomers frequently confront additional obstacles, including language barriers, cultural adaptation, social exclusion, and limited access to essential services (López-de-León et al., 2025; Verelst et al., 2022). For refugees, these challenges are often compounded by financial hardship and the complex psychosocial adjustments required by forced resettlement (Nguyen et al., 2024). Nonetheless, with appropriate support, many migrants successfully navigate these challenges, contributing meaningfully to their host communities. Psychological research indicates that effective coping strategies, social support networks, and a sense of belonging are key factors in promoting wellbeing and facilitating integration (Allen et al., 2017; Santini et al., 2020; Olcese et al., 2024; Song et al., 2025).

Social engagement within the host community is critical for integration (Penninx et al., 2008). Sport and leisure activities, in particular, have been shown to foster community connections, enhance belonging, and support both physical and psychological health (Spaaij, 2015; Donnelly and Coakley, 2002; McDonald et al., 2018; Walseth and Fasting, 2004). Leisure is typically understood as voluntary activities undertaken for enjoyment or personal fulfillment, ranging from physical exercise to recreational and social pursuits (Bum et al., 2021; Floyd, 1997). However, participation can be limited or even alienating when structural barriers—such as financial costs, logistical constraints, or cultural exclusion—remain unaddressed (Mohammadi, 2019; Gobster, 2002; Lovelock et al., 2011; Stodolska, 2015).

In Canada, and particularly in Montréal, research on migrants’ engagement with sport and leisure is limited, despite the city’s large and diverse newcomer population. Access to activities varies considerably: low-cost options such as walking, jogging, and soccer are widely accessible, whereas swimming, gym participation, and other organized programs often require financial resources, equipment, or administrative navigation. Seasonal weather further restricts outdoor activities and increases reliance on indoor programs (Adjizian et al., 2021; Barbo and Jeeroburkhan, 2025). Participation also varies according to gender, nationality, and systemic access barriers, though sport can strengthen a sense of belonging and facilitate social integration (Djogbenou et al., 2025a; Djogbenou et al., 2025b).

Building on these insights, this study examines the relationship between sport and leisure participation and the settlement experiences of newly arrived refugees and immigrants in Montréal. It addresses three research questions:

1. How do newly arrived immigrants and refugees engage with sport and leisure activities after arrival?
2. What barriers and challenges do they encounter in accessing these opportunities?
3. How do sport and leisure activities influence their integration, wellbeing, and sense of belonging?

The study conceptualizes psychological wellbeing, inclusion, belonging, and adaptation as relational, everyday experiences rather than clinical constructs. *Inclusion* refers to opportunities for participation in social, linguistic, and community activities; *belonging* encompasses feelings of recognition and connection within these environments; *psychological* wellbeing reflects perceptions of safety, reduced stress, and stability; and *adaptation* denotes the ongoing process of adjusting to new social, cultural, and linguistic contexts. These concepts guide the interpretation of participants’ accounts without aiming to quantify outcomes or establish causal links between sport and leisure participation and specific measures of integration or wellbeing.

## 2 Literature review

### 2.1 Migration and integration in the Canadian context

Canada has long established itself as a global leader in immigration and refugee resettlement, underpinned by policies that emphasize multiculturalism and social inclusion (Atak et al., 2023). Since the formal adoption of multiculturalism in 1971—later reinforced by the Canadian Multiculturalism Act of 1988 (Tierney, 2007)—this principle has remained central to Canada’s national identity and integration policies (Winter, 2015; Kymlicka, 2020).

Despite this policy framework, integration remains uneven. Migrants continue to face barriers related to language acquisition, employment, racial segregation, and limited access to social networks (Mirchandani and Bromfield, 2021; Ghadi et al., 2023; Hanley et al., 2024). Recent studies emphasize that integration is shaped not only by official policies but also by everyday experiences and meaningful community connections (Preston et al., 2022), including in the realm of sports and leisure activities (Antoun and Karlis, 2025; Khan et al., 2018).

Based on the most recent Statistics Canada census data currently available, Montréal is one of the Canada’s major immigrant-receiving cities, with approximately one-third of its residents born outside the country (Statistics Canada, 2022), Between July 2023 and July 2024, the city’s population grew by over 91,000 residents—a 4.2% increase—representing one of the highest regional growth rates in Québec’s history. This surge was primarily driven by temporary immigration, such as international students, temporary foreign workers, and asylum seekers. Notably, non-permanent residents outnumbered newly admitted permanent residents across all regions (Institut de la statistique du Québec, 2024).

As Québec’s largest city, Montréal is officially French-speaking, in alignment with the province’s sole official language policy. However, it also functions as a bilingual metropolis with a substantial English-speaking community. This linguistic duality presents distinct challenges for newcomers, who must often navigate predominantly French-speaking institutions and social environments while simultaneously engaging with English-speaking networks (Gyan et al., 2023).

Although multiculturalism is widely embraced, Montréal, like many cities, is not immune to social inequalities. Recent Québec legislation, particularly Bills 21 and 96, has disproportionately impacted immigrant communities, compounding existing challenges. Bill 21, enacted in 2019, bans public sector employees in authority roles from wearing religious symbols, disproportionately impacting religious minorities—particularly Muslim women wearing hijabs. (Mégret, 2022). Bill 96, introduced in 2021, reinforces French language requirements, restricting access to English-language services and education, thus creating additional barriers for non-Francophone newcomers (Tekin and Trofimovich, 2024). Together, these laws have deepened social exclusion and amplified difficulties faced by vulnerable groups (Butler et al., 2021; Taher et al., 2024).

Sport and leisure activities are central to Canada’s national identity, reflecting values such as teamwork, fairness, and diversity (Joseph et al., 2012). Beyond their cultural significance, sports programs provide accessible spaces for newcomers to develop language skills, build social networks, and facilitate community integration by reducing participation barriers (Lauckner et al., 2022; Nadeau et al., 2016). Participation in such activities has been positively associated with enhanced mental health, effective coping strategies, and the reconstruction of social identity, particularly among refugees (Scherer et al., 2024).

Despite these benefits, research has highlighted a persistent disconnect between the ideals of inclusion and the lived experiences of migrants in Canadian contexts. High participation costs and limited free time during the resettlement period often restrict opportunities to engage in sport and leisure activities (Curtin et al., 2016). In addition, linguistic and cultural barriers within host communities may further exacerbate social exclusion and hinder meaningful participation (Japaridze and Kaplan, 2023).

In response, several Canadian cities with high concentrations of newcomers have implemented community-based sport initiatives tailored to migrant populations (Gagliardi et al., 2022). These programs frequently combine physical activity with broader settlement supports, such as mentorship opportunities and employment training. In Québec, for example, youth and low-income migrants benefit from subsidies that help offset transportation and registration costs (Canadian Heritage, 2016; Sport for Life Society, 2025). When integrated within comprehensive settlement strategies, such initiatives highlight the potential of sport as a vehicle for social inclusion. Moreover, they foster psychological resilience, self-esteem, and adaptive coping—critical assets for newcomers navigating the stresses of early resettlement (Kaya et al., 2022; Sjögren Forss et al., 2021).

However, current scholarship offers limited insight into how migrants experience sport and leisure across different stages of settlement, leaving a notable gap in understanding (Gyan et al., 2023; Spaaij et al., 2019). Research in the Canadian context has predominantly focused on youth participation, providing little understanding of the distinct needs, experiences, and perspectives of adult migrants during the early stages of resettlement (Robinson et al., 2019). This study addresses this gap by examining how engagement in sport and leisure activities shapes adult newcomers’—and their families’—sense of belonging, integration, and coping within the city of Montréal.

## 3 Methods

Data were collected through semi-structured interviews with 15 participants, comprising 12 refugees and three recent immigrants. This composition reflects the study’s primary focus on refugees while also providing comparative insights into the experiences of newcomers who migrated through different pathways. Following the provision of informed consent, each participant completed a brief, paper-based demographic questionnaire. The primary data—derived from the semi-structured interviews—addressed research questions outlined in the introduction, focusing particularly on sport and leisure as resources for migrant integration, allowing for in-depth, informal discussions on this topic. All interviews were conducted by the principal investigator, who was solely responsible for data collection. Importantly, the lead researcher had no prior relationship with the participants, and the interviews were conducted using a community-based approach designed to minimize power imbalances and foster open, candid dialogue (Wallerstein et al., 2019, pp. 20–23).

### 3.1 Participant recruitment and data collection

Participants were recruited using criterion sampling, a purposive method targeting individuals who met specific eligibility criteria: at least 18 years old, residing in Montréal, and having lived in Canada for approximately 4 months to 1 year. Recruitment materials, prepared by the lead researcher, were posted at partner organizations, including the YMCA (Cartierville), CACI (Centre d’aide aux immigrants et réfugiés), The Refugee Centre (Le Centre de Réfugiés), and Reeves Passion. Permission to conduct the study and recruit participants was obtained in advance from each organization through an introductory letter outlining the study’s purpose, introducing the research team, and specifying the permissions required. Interested individuals contacted the lead researcher directly, received detailed information about the study, and provided informed consent before participating.

All interviews were conducted in person, face-to-face, in accessible public spaces such as parks and cafés in downtown Montréal. These locations were chosen to create a relaxed and familiar environment where participants could feel comfortable, while also supporting privacy and minimizing disruptions. Interviews were audio-recorded and later transcribed to ensure accuracy and facilitate detailed analysis. English was the primary language, reflecting participants’ comfort and preference, although many were also fluent in French. Some participants were native English speakers, while others used English as an additional language. Although all participants could communicate without an interpreter, using a non-native language may have affected the nuance or depth of their responses. To address this, the interviewer employed slower speech, flexible phrasing, and clarification prompts when necessary.

A detailed interview guide (Appendix A) was used to structure conversations, featuring open-ended and probing questions to draw out participants’ experiences and perspectives. While interviews followed a conversational and largely unstructured format, the interviewer ensured that all key themes were addressed (Rubin and Rubin, 2011). Terms such as “mental wellbeing” and “integration” were not formally defined, allowing participants to describe their own interpretations. Follow-up prompts were used when needed to clarify meaning, recognizing that such concepts can differ across cultural and linguistic contexts. Psychological wellbeing and adaptation were explored qualitatively through participants’ narratives rather than standardized measures, in line with qualitative approaches emphasizing meaning-making over clinical assessment (Braun and Clarke, 2006, pp. 82, 84, 90).

Data collection continued until thematic saturation was reached, defined as the point at which no new concepts or themes emerged (Hennink and Kaiser, 2022). Saturation was achieved by the fifteenth interview, establishing a final sample of 15 participants, including 12 men and 3 women. While this gender imbalance reflects common recruitment challenges in migrant research and may limit diversity of perspectives, similar insights were observed across participants regarding the opportunities and potential of sport and leisure programs to support resettlement.

To honor participants’ time and contribution, an unconditional $50 cash honorarium was provided. Unlike more paternalistic forms of reimbursement, such as restricted gift cards, this approach respected participants’ autonomy, allowing them to use the funds freely without judgment or constraints (Scherer et al., 2025, p. 6).

To protect confidentiality, all participants were assigned pseudonyms, and identifying details were removed from transcripts and reporting to ensure anonymity. The study adhered to ethical principles outlined in the Declaration of Helsinki, and ethical approval was obtained from the University’s Research Ethics Board.

### 3.2 Participant profiles

Participants’ profiles shown in Table 1.

**Table 1:** Participants’ profiles.

Age	Gender	Origin	Immigration status	Sports & leisure activities involved
19 years	Male	India	Immigrant	Gym, soccer, walking, jogging
20 years	Male	Afghanistan	Immigrant	Soccer
23 years	Male	India	Immigrant	Basketball, walking
24 years	Female	Mexico	Refugee	Climbing, dog walking
31 years	Male	Mexico	Refugee	Climbing, running
32 years	Male	Syria	Refugee	Walking, cycling, gym, swimming
33 years	Male	Ghana	Refugee	Walking, cycling
35 years	Male	Iran	Refugee	Soccer, volleyball, swimming, cycling
38 years	Female	Denmark	Refugee	Walking, gym free events in the city
38 years	Male	Ghana	Refugee	Jogging, walking, soccer
40 years	Male	Bangladesh	Refugee	Walking, cycling
42 years	Female	Belarus	Refugee	Skating, walking, running, hiking
44 years	Male	Nigeria	Refugee	Soccer, jogging, walking
46 years	Male	Ghana	Refugee	Walking, cycling
53 years	Male	Ghana	Refugee	Walking

### 3.3 Data analysis

A primary thematic analysis was conducted following the approach outlined by Maykut and Morehouse (2002). Transcripts were manually coded using a systematic, iterative process to identify meaningful passages, which were then grouped into broader themes aligned with the study’s objectives.

To enhance transparency, an inductive line-by-line coding process was employed. The first author generated open codes by identifying significant segments across all transcripts, focusing on participants’ descriptions of barriers, exclusion, sport participation, and integration. Examples of initial codes included “job-seeking pressure,” “no time because of settlement tasks,” “winter too cold,” “cannot communicate in French,” “locals not welcoming,” “sports help with French learning,” and “physical activity prevents depression.” These codes were compared across interviews and grouped into broader conceptual categories using constant comparison.

Through iterative refinement, these categories were synthesized into three main themes and associated subthemes presented in the Findings:

1. Socio-economic and structural barriers to leisure and sport participation—including competing settlement demands, job-seeking pressures, and Montréal’s cold climate.
2. Experiences of social exclusion and marginalization in leisure spaces—encompassing communication difficulties due to language barriers and feelings of being marginalized as foreigners.
3. Combining sports and language learning to support inclusion and wellbeing.

It is important to note that the distinction between these three themes is not always clear-cut. Rather than being mutually exclusive, the themes should be understood as mutually reinforcing, reflecting the complex and intertwined ways in which structural barriers, social exclusion, and the potential benefits of sport and leisure interact in participants’ experiences.

Coding decisions and theme frameworks were reviewed collaboratively by the research team, with discrepancies resolved through consensus to ensure reliability and trustworthiness. This inductive approach allowed themes to emerge organically from the data without reliance on qualitative analysis software. Relevant theoretical and empirical literature was integrated to contextualize and interpret the findings.

Participants did not report direct or clinically evaluated improvements in psychological wellbeing, identity, or belonging from their participation in sport and leisure. Instead, their responses highlighted perceived challenges and the practical, context-dependent ways they believed sport and leisure might support language learning and social connection during settlement.

## 4 Findings

### 4.1 Socio-economic and structural barriers to leisure and sport participation

One key theme that emerged from our interviews highlights the socio-economic and structural barriers that limited participants’ access to leisure and sport activities. Key sub-themes included the competing demands of settlement, the pressure of job-seeking, and the impact of Montréal’s cold climate.

In the early stages of resettlement, participants reported focusing primarily on meeting basic needs such as securing housing, obtaining winter clothing, and navigating the city. As Lindsay (female, 38, Denmark) explained:

It’s hard to think about things like sports when you’re just trying to figure out where to get winter clothes or how the metro works.

Similarly, Paulette (female, 24, Mexico) shared:

At the beginning, I didn’t want to go out. I didn’t have sports shoes. I didn’t have anyone to talk to. My house felt like the only safe space.

For some, the cost of sport activities acted as a significant barrier. Ossian (male, 31, Mexico) noted the mental health impact of being unable to afford preferred activities:

If I don't do sports, I'm going to kill myself, for sure. Okay, yeah, because before doing rock climbing, it's expensive. It's super, super expensive, and we didn't have any money to pay. So, when we just arrived here, like, one month after we arrived here, I told my wife, I need to do something, because I'm going to get crazy. So, I started running.

Participants who were unemployed or underemployed described how job-seeking responsibilities took precedence over leisure. Ossian (male, 31, Mexico) added:

If we had jobs, we could afford it [climbing], but right now it’s impossible.

Kylan (male, 23, India) echoed this sentiment:

Depending on how many classes I have and how many assignments… if you’re also looking for a job, there’s just no time for anything else.

Montréal’s harsh winter climate was frequently mentioned as a barrier to outdoor activity. Farid (male, 20, Afghanistan) explained:

I used to do mountain biking in Afghanistan, but I cannot continue here because of the weather. It is way too cold.

Hamid (male, 32, Iran) added:

It’s cold, roads are full of snow. Soccer is only played during the summer here. In my country, we could play all year.

These findings underscore how the interplay of settlement demands, financial limitations, employment pressures, and environmental conditions can severely constrain newcomers’ engagement in sport and leisure, particularly during the initial stages of resettlement. Although all participants expressed a desire to participate in such activities, they emphasized the significant barriers to accessing them. They further stressed that these challenges cannot be resolved through programming alone, as the structural constraints they encountered were far more entrenched—ranging from a lack of time and financial resources for job-seeking newcomers to the inclement weather that forced many, particularly those from warmer climates, to radically rethink the outdoor activities they once enjoyed year-round at low cost, such as soccer. These reflections mirror the cultural and identity-based constraints discussed in the following section.

### 4.2 Experiences of social exclusion and marginalization in leisure spaces

Another key theme that emerged from our interviews was that, despite Montréal’s multicultural identity, many participants experienced exclusion from mainstream social and sporting circles. Participants described a range of experiences that left them feeling marginalized within the city’s sport and leisure environments. These experiences appeared to arise from two interconnected mechanisms: difficulties communicating in French and a sense of being treated as outsiders or foreigners.

Many participants explained that limited proficiency in French or English constrained their ability to communicate, follow instructions, and form social connections. Saad (male, 33, Ghana) remarked:

I cannot communicate with them. They don’t speak English—just French. That’s why I cannot play sports here.

Ossian (male, 31, Mexico) described similar struggles in sport settings:

Even at the front desk, it was hard to say I want to climb or ask how to pay. They ended up speaking English, but it was a struggle.

Hamid (male, 32, Iran) summarized the broader impact of language barriers:

There are some language barriers. You cannot communicate with the people because you are not fluent in the language. So, it is hard.

While language emerged as the most prominent challenge, several participants also described forms of exclusion unrelated to linguistic ability, including cultural distance, perceived discrimination, and a general lack of openness from established groups. Even when participants could communicate effectively, mainstream leisure spaces often felt difficult to access or subtly unwelcoming, reinforcing broader feelings of social exclusion.

Salah (male, 40, Bangladesh) shared:

You know that I am an outsider. So, the native people may not be very friendly with me in terms of taking me to play with them.

Paulette (female, 24, Mexico) echoed this sense of social distance:

In Mexico, if someone’s new, you say hi and help them. Here, it’s like everyone is in their own world.

Many participants found it easier to connect with other immigrants or racialized individuals, particularly those who shared similar migration experiences. Paulette (female, 24, Mexico) explained:

If I feel vulnerable, I don’t want to be around someone who might hate me. I stay close to immigrant or racialized people who understand.

Kylan (male, 23, India) added:

We don’t see many Québecois in our sports spaces. Most people I talk to are other immigrants.

Overall, these findings highlight how language and social barriers intersect to shape immigrants’ participation in Montréal’s sport and leisure environments. These barriers often limited access to mainstream spaces and encouraged reliance on ethnically or culturally similar networks. Notably, while language difficulties were central, many participants emphasized a deeper feeling of unwelcomeness—particularly among racialized newcomers—which pushed them to remain close to those who shared similar backgrounds. This tendency, though understandable, may inadvertently heighten social separation and slow integration. Thus, although sport and leisure have the potential to serve as bridges between newcomers and the host community, they can also reinforce exclusion when newcomers perceive—or experience—a lack of openness or willingness to include them.

### 4.3 Combining sports and language learning to support inclusion and wellbeing

The final theme from our interviews draws attention to participants’ interest in integrating sports and leisure with French-language learning—a particularly relevant possibility given recent changes in Québec’s immigration policy. Starting December 17, 2025, foreign workers who apply under the Temporary Foreign Worker Program (TFWP) and who have accumulated 3 years of work experience in Québec will need to demonstrate spoken-French proficiency at level 4 (NCLC) to qualify for a renewed work permit (CIC News, 2025). In addition, permanent-immigration streams under the Québec Experience Program (PEQ) and other economic-immigration programs increasingly require defined levels of French as part of eligibility (Gouvernement du Québec, n.d.). This regulatory context strengthens the practical importance of combining francization with daily life—including sport and leisure—rather than leaving language learning confined to classrooms.

Many participants suggested that sport and leisure programs could be intentionally structured to include francization, making language acquisition more enjoyable, accessible, and socially meaningful. In this conception, sport and leisure function not only as recreational outlets, but also as important tools for language development and social connection during resettlement.

Lindsay (female, 38, Denmark) succinctly expressed this idea:

Doing sports also helps you learn French… because they are speaking French. It would be good and healthy.

Paulette (female, 24, Mexico) proposed a more structured approach:

We spend all day in francization classes. Why not make one hour more interactive, like soccer in French?

Kylan (male, 23, India) connected this concept to broader integration goals:

Universities mix sport and culture all the time. Why can’t the government do the same for refugees? It helps you find others in the same situation.

Salah (male, 40, Bangladesh) emphasized the linguistic benefits:

In French courses, sports can be a part of a course or integrated with a French course.

In general, participants observed that sport and leisure settings can provide informal, low-pressure spaces where language learning and social connections emerge naturally. They described how physical activity encourages communication, collaboration, and shared routines, making French practice feel more approachable and engaging than in classroom settings. Many reflected that, under the right conditions, combining sport with language learning could help newcomers feel included and support their integration in a predominantly French-speaking society. Participants emphasized that beyond recreation, these activities offer opportunities to build relationships, practice French, and develop a sense of belonging, helping them navigate resettlement challenges while forming meaningful, long-term connections.

## 5 Discussion

Scholars in Immigrant and Refugee Studies emphasize the central role of social integration in resettlement, noting its impact on health, wellbeing, and overall quality of life (Cheung and Phillimore, 2013; Correa-Velez et al., 2010; Fozdar and Hartley, 2013; Valtonen, 1998). According to Berry (1997), successful integration occurs when migrants maintain their cultural identity while accessing the resources necessary for economic self-sufficiency and full participation in social life. Integration is therefore a two-way process, requiring engagement from both newcomers and host communities to foster social bonds and positive identities (Spaaij, 2015; Walseth and Fasting, 2004; Baumeister and Leary, 1995).

This study examined the role of sport and leisure in the resettlement of newly arrived refugees in Montréal, a highly bilingual city where nearly one-third of residents are foreign-born (Statistics Canada, 2022). Participants from non-Francophone countries identified French proficiency as a major barrier, consistent with research linking language skills to reduced acculturative stress and improved wellbeing (Kaufmann, 2021; Khan et al., 2018). Similarly, French-speaking migrants learning English faced linguistic challenges, highlighting the bidirectional nature of language barriers (Gyan et al., 2023; de Porto Oliveira and Gosselin, 2024). Language difficulties extended into leisure contexts, reinforcing perceptions of outsider status and, in some cases, subtle discrimination.

Participants described sport and leisure as informal, low-pressure spaces where communication, collaboration, and shared routines naturally occur, making language practice feel more accessible and socially meaningful than classroom instruction. They noted that physical activity helped manage stress, establish routines, and create a sense of normalcy during resettlement, even when formal supports were limited. Yet, socio-economic constraints, program inaccessibility, and cultural misalignment often prevented participation, despite recognition of potential benefits.

Social identity theory (Tajfel and Turner, 2004) helps explain these dynamics: many participants identified more strongly with other migrant or racialized communities, which provided psychological protection and resilience through solidarity networks (Brance et al., 2024; Ungar, 2011; Gyan et al., 2023). Evidence from Canada and Sweden indicates that sports participation can enhance coping and social connectedness among migrants (Kaya et al., 2022; Sjögren Forss et al., 2021), and refugee football programs have been linked to improved mental health and sense of belonging (Scherer et al., 2024). In Montréal, informal activities such as running were low-barrier and accessible, whereas activities like rock climbing were limited by cost or logistics, highlighting the importance of flexible, inclusive programming.

Gender shaped participation as well. With only three women in the sample, the findings reflect persistent barriers for women, including safety concerns, cultural expectations, and caregiving responsibilities, which intersected with language and socio-economic constraints to further limit access. While the sample size limits systematic comparisons across refugee subgroups, preliminary observations suggest that participants’ cultural backgrounds, language skills, and prior experience with organized sports influenced how easily they engaged with Montréal’s sport and leisure system. These observations, although preliminary, point to meaningful avenues for future research on the intersection of gender, culture, and prior sports experience in shaping participation.

Integrating language learning into sport emerged as a particularly promising strategy. Participants observed that physical activity encourages communication, teamwork, and shared routines, creating informal contexts for practicing French. This approach simultaneously addresses linguistic and psychosocial barriers, complementing formal francization programs while supporting broader social integration. Community-led initiatives combining recreation and settlement services have shown promise, and frameworks such as Sport for Life provide adaptable models for inclusive programming (Doidge et al., 2020; Gagliardi et al., 2022; Robinson et al., 2019; Hermens et al., 2017).

Despite Canada’s reputation for inclusivity (Atak et al., 2023; Winter, 2015), participants’ experiences reveal persistent structural barriers, including affordability, time constraints, and insufficiently inclusive programming. Addressing these challenges requires subsidized access, targeted outreach, and facilitator training to counter exclusionary dynamics. Embedded within integration services, sport and leisure can serve as psychosocial resources, reducing stress, fostering connection, and supporting identity reconstruction. Leisure spaces thus function not only as sites of recreation but also as critical infrastructures for resilience and social integration (Ungar, 2011; Sia et al., 2020; Borges et al., 2023; Spaaij et al., 2019).

Participants highlighted that combining sport with language learning could help newcomers feel included and supported in a predominantly French-speaking society. Beyond recreation, these activities provide flexible tools for building relationships, practicing French, and cultivating a sense of belonging, helping newcomers navigate resettlement challenges while forming meaningful, long-term connections. These observations reinforce the dual potential of sport and leisure: as spaces for psychosocial support and as practical, adaptable platforms for social and linguistic integration. By reducing barriers, fostering inclusion, and providing structured yet flexible opportunities, sport and leisure can play a central role in helping newcomers establish networks, build resilience, and participate fully in their new communities.

## 6 Conclusion

Sports and leisure activities are widely recognized as valuable resources for supporting newcomers’ integration, promoting physical and mental health, fostering social interaction, and enabling cultural exchange (Doidge et al., 2020; Spaaij et al., 2019). Reflecting this, many countries have implemented programs aimed at enhancing both the wellbeing and socio-cultural integration of migrant populations (Agergaard, 2018; Hoye et al., 2015; Olliff, 2008; Sherry et al., 2015; Sjögren Forss et al., 2021). By centering newcomers’ own observations, this study highlights their perspectives, offering insights often overlooked in institution-focused research.

In Montréal, participants noted the paradoxical role of sport and leisure: while they observed exclusion arising from socio-economic constraints and language barriers, they also saw ways in which these activities could support settlement. For instance, sport and leisure might provide informal opportunities for practicing French, establishing routines, or connecting with others who share similar migration experiences. Rather than acting as direct pathways to improved wellbeing or social belonging, these activities function as complementary social resources within broader integration strategies, highlighting their potential rather than guaranteed effects.

Limitations of this study should be considered. The sample was gender-imbalanced (12 men, 3 women), which restricts understanding of women’s distinct experiences and barriers. The study was conducted exclusively in Montréal, a highly bilingual and culturally diverse city, potentially limiting generalizability to other regions. Additionally, the study is based on participants’ observations rather than direct measures of psychosocial outcomes, so the benefits identified remain potential rather than empirically confirmed.

Future research should address these limitations by exploring gendered and intersectional experiences of sport and leisure, employing longitudinal designs to assess the long-term psychosocial impacts of participation, and conducting comparative studies across different Canadian contexts. Mixed-methods approaches could also enhance the robustness of findings by triangulating qualitative insights with quantitative measures of wellbeing, language acquisition, and social integration—ultimately offering a more comprehensive understanding of how sport and leisure contribute to the settlement experience.

## Data Availability

The datasets presented in this article are not readily available because the datasets generated and analyzed during this study are not publicly available due to the sensitive nature of the qualitative interview transcripts, which may contain potentially identifiable information. In accordance with the ethical approval granted by the institutional research ethics board and to protect participant confidentiality, full transcripts cannot be shared publicly. Requests for data access may be directed to the appropriate research ethics board, subject to ethical and institutional approval. Requests to access the datasets should be directed to https://www.mcgill.ca/research/research/human/contact-us.

## Appendix A: Semi-structured interview guide

The semi-structured interview guide was developed to explore newcomers’ experiences with sport and leisure activities in Quebec, focusing on challenges, motivations, and psychosocial outcomes. The interviews comprised six sections and used open-ended questions to encourage detailed responses.

1. Background Information

◦ How long have you been in Canada, and what was your job or daily routine before you arrived?
◦ How does the Canadian climate impact your daily life and mobility, and what is your experience?

2. Sport and Leisure History

◦ What kinds of sports or leisure activities did you engage in prior to relocating to Canada?
◦ Are you still engaged in those activities here, or have your interests shifted since you arrived?

3. Barriers and Challenges

◦ What difficulties have you encountered when participating in sport or leisure activities (e.g., cost, time, language, facilities)?
◦ How do social or cultural differences influence your ability to participate?

4. Motivations and Expectations

◦ What motivates you to take part in sport and leisure activities?
◦ What are your expectations of these programs in Quebec?

5. Perceived Benefits

◦ How does participation in sport or leisure affect your mental wellbeing, sense of belonging, or social life?
◦ Do you see these activities as helping with integration into Canadian society?

6. Recommendations and Final Reflections

◦ What could be improved to make sport and leisure more accessible for newcomers?
◦ Is there anything else you would like to share about your experiences?

Note: The guide served as a flexible framework; follow-up and probing questions were used as needed.
